# *N*-(2,6-Dimethyl­phen­yl)benzene­sulfonamide

**DOI:** 10.1107/S1600536808024653

**Published:** 2008-08-06

**Authors:** B. Thimme Gowda, Sabine Foro, K. S. Babitha, Hartmut Fuess

**Affiliations:** a Department of Chemistry, Mangalore University, Mangalagangotri 574 199, Mangalore, India; b Institute of Materials Science, Darmstadt University of Technology, Petersenstrasse 23, D-64287 Darmstadt, Germany

## Abstract

In the crystal structure of the title compound, C_14_H_15_NO_2_S, the N—H bond is *trans* to one of the S=O double bonds, similar to what is observed in *N*-(2-methyl­phen­yl)benzene­sulfonamide and other aryl sulfonamides. The two aromatic rings enclose a dihedral angle of 44.9 (1)°. The mol­ecules are connected by inter­molecular N—H⋯O hydrogen bonds into chains running along the *a* axis. An intermolecular C—H⋯O hydrogen bond is also present.

## Related literature

For related literature, see: Gelbrich *et al.* (2007 ▶); Gowda *et al.* (2005 ▶, 2008 ▶); Gowda, Babitha *et al.* (2007 ▶); Gowda, Foro *et al.* (2007 ▶); Perlovich *et al.* (2006 ▶).
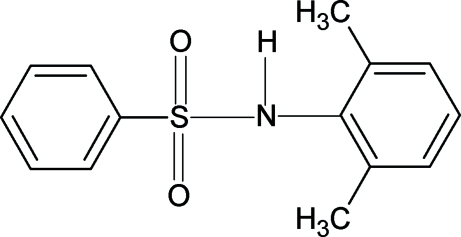


## Experimental

### 

#### Crystal data

C_14_H_15_NO_2_S
*M* *_r_* = 261.33
Monoclinic, 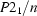

*a* = 5.2133 (7) Å
*b* = 17.971 (2) Å
*c* = 14.040 (1) Å
β = 91.681 (9)°
*V* = 1314.8 (2) Å^3^
*Z* = 4
Cu *K*α radiation
μ = 2.13 mm^−1^
*T* = 299 (2) K
0.55 × 0.35 × 0.33 mm

#### Data collection

Enraf–Nonius CAD-4 diffractometer
Absorption correction: none
2622 measured reflections
2340 independent reflections
2197 reflections with *I* > 2σ(*I*)
*R*_int_ = 0.071
3 standard reflections frequency: 120 min intensity decay: 1.0%

#### Refinement

*R*[*F* ^2^ > 2σ(*F* ^2^)] = 0.047
*wR*(*F* ^2^) = 0.134
*S* = 1.08
2340 reflections
164 parameters
H-atom parameters constrained
Δρ_max_ = 0.35 e Å^−3^
Δρ_min_ = −0.41 e Å^−3^

### 

Data collection: *CAD-4-PC* (Enraf–Nonius, 1996 ▶); cell refinement: *CAD-4-PC*; data reduction: *REDU4* (Stoe & Cie, 1987 ▶); program(s) used to solve structure: *SHELXS97* (Sheldrick, 2008 ▶); program(s) used to refine structure: *SHELXL97* (Sheldrick, 2008 ▶); molecular graphics: *PLATON* (Spek, 2003 ▶); software used to prepare material for publication: *SHELXL97*.

## Supplementary Material

Crystal structure: contains datablocks I, global. DOI: 10.1107/S1600536808024653/bt2760sup1.cif

Structure factors: contains datablocks I. DOI: 10.1107/S1600536808024653/bt2760Isup2.hkl

Additional supplementary materials:  crystallographic information; 3D view; checkCIF report

## Figures and Tables

**Table 1:** Hydrogen-bond geometry (Å, °)

*D*—H⋯*A*	*D*—H	H⋯*A*	*D*⋯*A*	*D*—H⋯*A*
N1—H1*N*⋯O1^i^	0.86	2.55	3.179 (2)	131
C5—H5⋯O2^ii^	0.93	2.57	3.229 (3)	129

Symmetry codes: (i) 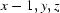; (ii) 
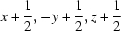
.

**Fig. 1.**
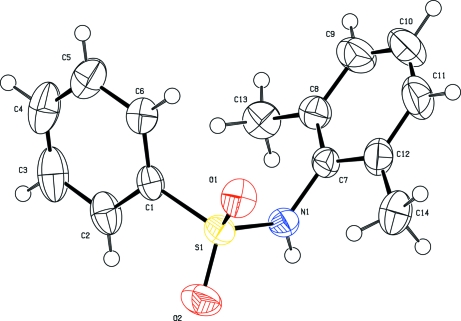
Molecular structure of the title compound, showing the atom labeling scheme. The displacement ellipsoids are drawn at the 50% probability level. The H atoms are represented as small spheres of arbitrary radii.

**Fig. 2.**
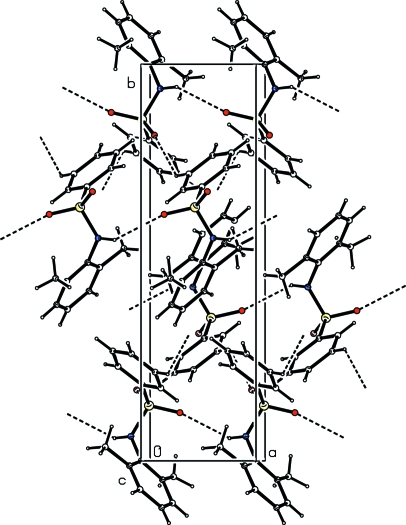
Molecular packing of the title compound with hydrogen bonding shown as dashed lines.

## supplementary crystallographic information

### Comment

As part of a study of the substituent effects on the crystal structures of *N*-(aryl)-sulfonamides, in the present work, the structure of *N*-(2,6-dimethylphenyl)-benzenesulfonamide (N26DMPBSA) has been determined (Gowda *et al.*, 2005, 2008; Gowda, Babitha *et al.* 2007; Gowda, Foro *et al.* 2007). The the N—H bond is trans to one of the S—O double bonds (Fig. 1), similar to what is observed in *N*-(2-methylphenyl)-benzenesulfonamide (N2MPBSA)(Gowda *et al.*, 2008) and other aryl sulfonamides (Perlovich *et al.*, 2006; Gelbrich *et al.*, 2007;). The two aromatic rings are rotated relative to each other by 44.9 (1)°, compared with the value of 61.5 (1)° in N2MPBSA. The other bond parameters in N26DMPBSA are similar to those observed in N2MPBSA and other *N*-(aryl)-sulfonamides (Gowda, Babitha *et al.*, 2007; Gowda, Foro *et al.* 2007; Gowda *et al.* 2008; Perlovich *et al.*, 2006; Gelbrich *et al.*, 2007). The packing diagram of N26DMPBSA *via* intermolecular N—H···O and C—H···O hydrogen bonds (Table 1) is shown in Fig. 2.

### Experimental

The solution of benzene (10 cc) in chloroform (40 cc) was treated dropwise with chlorosulfonic acid (25 cc) at 0 ° C. After the initial evolution of hydrogen chloride subsided, the reaction mixture was brought to room temperature and poured into crushed ice in a beaker. The chloroform layer was separated, washed with cold water and allowed to evaporate slowly. The residual benzenesulfonylchloride was treated with 2,6-dimethylaniline in the stoichiometric ratio and boiled for ten minutes. The reaction mixture was then cooled to room temperature and added to ice cold water (100 cc). The resultant solid *N*-(2,6-dimethylphenyl)-benzenesulfonamide was filtered under suction and washed thoroughly with cold water. It was then recrystallized to constant melting point from dilute ethanol. The purity of the compound was checked and characterized by recording its infrared and NMR spectra (Gowda *et al.*, 2005). The single crystals used in X-ray diffraction studies were grown in ethanolic solution by slow evaporation at room temperature.

### Refinement

The H atoms were positioned with idealized geometry using a riding model with C—H = 0.93–0.96 Å, N—H = 0.86 Å, and were refined with isotropic displacement parameters (set to 1.2 times of the *U*_eq_ of the parent atom).

### Crystal data

**Table:**

C_14_H_15_NO_2_S	*F*_000_ = 552
*M**_r_* = 261.33	*D*_x_ = 1.320 Mg m^−^^3^
Monoclinic, *P*2_1_/*n*	Cu *K*α radiation λ = 1.54180 Å
Hall symbol: -P 2yn	Cell parameters from 25 reflections
*a* = 5.2133 (7) Å	θ = 4.0–19.2º
*b* = 17.971 (2) Å	µ = 2.14 mm^−^^1^
*c* = 14.040 (1) Å	*T* = 299 (2) K
β = 91.681 (9)º	Prism, colourless
*V* = 1314.8 (2) Å^3^	0.55 × 0.35 × 0.33 mm
*Z* = 4	

### Data collection

**Table:**

Enraf–Nonius CAD-4 diffractometer	*R*_int_ = 0.071
Radiation source: fine-focus sealed tube	θ_max_ = 66.9º
Monochromator: graphite	θ_min_ = 4.0º
*T* = 299(2) K	*h* = −6→1
ω/2θ scans	*k* = −21→0
Absorption correction: none	*l* = −16→16
2622 measured reflections	3 standard reflections
2340 independent reflections	every 120 min
2197 reflections with *I* > 2σ(*I*)	intensity decay: 1.0%

### Refinement

**Table:**

Refinement on *F*^2^	Hydrogen site location: inferred from neighbouring sites
Least-squares matrix: full	H-atom parameters constrained
*R*[*F*^2^ > 2σ(*F*^2^)] = 0.047	*w* = 1/[σ^2^(*F*_o_^2^) + (0.0807*P*)^2^ + 0.6081*P*] where *P* = (*F*_o_^2^ + 2*F*_c_^2^)/3
*wR*(*F*^2^) = 0.134	(Δ/σ)_max_ = 0.001
*S* = 1.08	Δρ_max_ = 0.35 e Å^−^^3^
2340 reflections	Δρ_min_ = −0.41 e Å^−^^3^
164 parameters	Extinction correction: SHELXL97 (Sheldrick, 2008), Fc^*^=kFc[1+0.001xFc^2^λ^3^/sin(2θ)]^-1/4^
Primary atom site location: structure-invariant direct methods	Extinction coefficient: 0.0060 (9)
Secondary atom site location: difference Fourier map	

### Special details

**Table:**

Geometry. All e.s.d.'s (except the e.s.d. in the dihedral angle between two l.s. planes) are estimated using the full covariance matrix. The cell e.s.d.'s are taken into account individually in the estimation of e.s.d.'s in distances, angles and torsion angles; correlations between e.s.d.'s in cell parameters are only used when they are defined by crystal symmetry. An approximate (isotropic) treatment of cell e.s.d.'s is used for estimating e.s.d.'s involving l.s. planes.
Refinement. Refinement of *F*^2^ against ALL reflections. The weighted *R*-factor *wR* and goodness of fit *S* are based on *F*^2^, conventional *R*-factors *R* are based on *F*, with *F* set to zero for negative *F*^2^. The threshold expression of *F*^2^ > σ(*F*^2^) is used only for calculating *R*-factors(gt) *etc*. and is not relevant to the choice of reflections for refinement. *R*-factors based on *F*^2^ are statistically about twice as large as those based on *F*, and *R*- factors based on ALL data will be even larger.

### Fractional atomic coordinates and isotropic or equivalent isotropic displacement parameters (Å^2^)

**Table:**

	*x*	*y*	*z*	*U*_iso_*/*U*_eq_	
C1	−0.0070 (4)	0.19125 (11)	0.78195 (15)	0.0359 (5)	
C2	−0.2175 (4)	0.23812 (13)	0.7860 (2)	0.0518 (6)	
H2	−0.3303	0.2434	0.7339	0.062*	
C3	−0.2557 (5)	0.27695 (15)	0.8699 (3)	0.0677 (8)	
H3	−0.3972	0.3081	0.8744	0.081*	
C4	−0.0875 (6)	0.26990 (16)	0.9461 (2)	0.0666 (8)	
H4	−0.1141	0.2969	1.0014	0.080*	
C5	0.1186 (6)	0.22354 (15)	0.94146 (19)	0.0590 (7)	
H5	0.2309	0.2186	0.9938	0.071*	
C6	0.1608 (4)	0.18376 (13)	0.85885 (17)	0.0453 (5)	
H6	0.3017	0.1522	0.8554	0.054*	
C7	−0.0178 (4)	0.00007 (11)	0.74093 (15)	0.0373 (5)	
C8	−0.1139 (4)	−0.01229 (12)	0.83144 (16)	0.0437 (5)	
C9	−0.0176 (6)	−0.07186 (15)	0.8839 (2)	0.0609 (7)	
H9	−0.0788	−0.0809	0.9444	0.073*	
C10	0.1675 (7)	−0.11784 (16)	0.8480 (3)	0.0730 (9)	
H10	0.2336	−0.1568	0.8848	0.088*	
C11	0.2541 (6)	−0.10620 (14)	0.7582 (2)	0.0680 (8)	
H11	0.3769	−0.1382	0.7343	0.082*	
C12	0.1628 (5)	−0.04751 (13)	0.70148 (18)	0.0498 (6)	
C13	−0.3213 (5)	0.03545 (14)	0.87194 (18)	0.0521 (6)	
H13A	−0.4740	0.0316	0.8323	0.063*	
H13B	−0.2651	0.0863	0.8741	0.063*	
H13C	−0.3570	0.0189	0.9352	0.063*	
C14	0.2511 (6)	−0.03980 (16)	0.6006 (2)	0.0683 (8)	
H14A	0.2690	0.0120	0.5853	0.082*	
H14B	0.1270	−0.0622	0.5576	0.082*	
H14C	0.4135	−0.0643	0.5947	0.082*	
N1	−0.1132 (3)	0.06164 (9)	0.68444 (12)	0.0371 (4)	
H1N	−0.2573	0.0571	0.6536	0.044*	
O1	0.3127 (3)	0.12146 (10)	0.67758 (12)	0.0478 (4)	
O2	−0.0692 (4)	0.17882 (10)	0.59926 (12)	0.0576 (5)	
S1	0.04474 (9)	0.13941 (3)	0.67748 (3)	0.0358 (2)	

### Atomic displacement parameters (Å^2^)

**Table:**

	*U*^11^	*U*^22^	*U*^33^	*U*^12^	*U*^13^	*U*^23^
C1	0.0330 (10)	0.0277 (9)	0.0476 (11)	−0.0044 (7)	0.0082 (8)	0.0010 (8)
C2	0.0383 (11)	0.0382 (12)	0.0794 (17)	0.0005 (9)	0.0079 (11)	−0.0024 (11)
C3	0.0519 (14)	0.0440 (14)	0.109 (2)	0.0011 (11)	0.0324 (16)	−0.0178 (14)
C4	0.0731 (18)	0.0538 (15)	0.0749 (19)	−0.0173 (14)	0.0336 (15)	−0.0237 (14)
C5	0.0697 (16)	0.0576 (15)	0.0500 (14)	−0.0172 (13)	0.0086 (12)	−0.0106 (12)
C6	0.0437 (11)	0.0425 (12)	0.0500 (12)	−0.0010 (9)	0.0044 (9)	−0.0046 (9)
C7	0.0377 (10)	0.0300 (10)	0.0437 (11)	−0.0023 (8)	−0.0068 (8)	−0.0014 (8)
C8	0.0421 (11)	0.0398 (11)	0.0490 (12)	−0.0076 (9)	−0.0027 (9)	0.0031 (9)
C9	0.0698 (17)	0.0511 (14)	0.0614 (16)	−0.0060 (12)	−0.0037 (12)	0.0180 (12)
C10	0.088 (2)	0.0455 (14)	0.085 (2)	0.0092 (14)	−0.0165 (17)	0.0183 (14)
C11	0.0701 (18)	0.0419 (14)	0.091 (2)	0.0179 (13)	−0.0075 (15)	−0.0061 (14)
C12	0.0523 (13)	0.0388 (12)	0.0579 (14)	0.0040 (10)	−0.0045 (10)	−0.0108 (10)
C13	0.0489 (13)	0.0557 (14)	0.0523 (13)	−0.0057 (11)	0.0106 (10)	0.0054 (11)
C14	0.0828 (19)	0.0587 (16)	0.0638 (17)	0.0143 (14)	0.0110 (14)	−0.0211 (13)
N1	0.0333 (8)	0.0372 (9)	0.0402 (9)	−0.0016 (7)	−0.0069 (7)	0.0004 (7)
O1	0.0331 (8)	0.0539 (9)	0.0569 (10)	−0.0006 (7)	0.0104 (7)	−0.0048 (7)
O2	0.0690 (11)	0.0563 (10)	0.0473 (9)	0.0011 (8)	−0.0016 (8)	0.0193 (8)
S1	0.0351 (3)	0.0364 (3)	0.0361 (3)	−0.00027 (18)	0.0040 (2)	0.00487 (18)

### Geometric parameters (Å, °)

**Table:**

C1—C6	1.376 (3)	C9—C10	1.377 (5)
C1—C2	1.386 (3)	C9—H9	0.9300
C1—S1	1.765 (2)	C10—C11	1.368 (5)
C2—C3	1.388 (4)	C10—H10	0.9300
C2—H2	0.9300	C11—C12	1.397 (4)
C3—C4	1.369 (5)	C11—H11	0.9300
C3—H3	0.9300	C12—C14	1.508 (4)
C4—C5	1.363 (4)	C13—H13A	0.9600
C4—H4	0.9300	C13—H13B	0.9600
C5—C6	1.386 (3)	C13—H13C	0.9600
C5—H5	0.9300	C14—H14A	0.9600
C6—H6	0.9300	C14—H14B	0.9600
C7—C8	1.397 (3)	C14—H14C	0.9600
C7—C12	1.398 (3)	N1—S1	1.6263 (17)
C7—N1	1.441 (3)	N1—H1N	0.8600
C8—C9	1.386 (3)	O1—S1	1.4334 (16)
C8—C13	1.505 (3)	O2—S1	1.4221 (17)
			
C6—C1—C2	120.9 (2)	C9—C10—H10	120.0
C6—C1—S1	119.41 (16)	C10—C11—C12	121.7 (3)
C2—C1—S1	119.71 (18)	C10—C11—H11	119.2
C1—C2—C3	118.3 (3)	C12—C11—H11	119.2
C1—C2—H2	120.8	C11—C12—C7	117.3 (3)
C3—C2—H2	120.8	C11—C12—C14	119.8 (2)
C4—C3—C2	120.8 (2)	C7—C12—C14	122.9 (2)
C4—C3—H3	119.6	C8—C13—H13A	109.5
C2—C3—H3	119.6	C8—C13—H13B	109.5
C5—C4—C3	120.5 (3)	H13A—C13—H13B	109.5
C5—C4—H4	119.8	C8—C13—H13C	109.5
C3—C4—H4	119.8	H13A—C13—H13C	109.5
C4—C5—C6	120.0 (3)	H13B—C13—H13C	109.5
C4—C5—H5	120.0	C12—C14—H14A	109.5
C6—C5—H5	120.0	C12—C14—H14B	109.5
C1—C6—C5	119.5 (2)	H14A—C14—H14B	109.5
C1—C6—H6	120.2	C12—C14—H14C	109.5
C5—C6—H6	120.2	H14A—C14—H14C	109.5
C8—C7—C12	121.8 (2)	H14B—C14—H14C	109.5
C8—C7—N1	119.69 (19)	C7—N1—S1	121.72 (13)
C12—C7—N1	118.5 (2)	C7—N1—H1N	119.1
C9—C8—C7	118.2 (2)	S1—N1—H1N	119.1
C9—C8—C13	119.5 (2)	O2—S1—O1	119.85 (11)
C7—C8—C13	122.3 (2)	O2—S1—N1	105.89 (10)
C10—C9—C8	121.0 (3)	O1—S1—N1	107.55 (10)
C10—C9—H9	119.5	O2—S1—C1	107.95 (11)
C8—C9—H9	119.5	O1—S1—C1	106.89 (10)
C11—C10—C9	120.0 (3)	N1—S1—C1	108.28 (9)
C11—C10—H10	120.0		
			
C6—C1—C2—C3	−0.4 (3)	C10—C11—C12—C14	−176.0 (3)
S1—C1—C2—C3	178.70 (18)	C8—C7—C12—C11	−3.2 (3)
C1—C2—C3—C4	0.9 (4)	N1—C7—C12—C11	179.3 (2)
C2—C3—C4—C5	−1.1 (4)	C8—C7—C12—C14	174.0 (2)
C3—C4—C5—C6	0.7 (4)	N1—C7—C12—C14	−3.5 (3)
C2—C1—C6—C5	0.1 (3)	C8—C7—N1—S1	99.9 (2)
S1—C1—C6—C5	−179.04 (18)	C12—C7—N1—S1	−82.5 (2)
C4—C5—C6—C1	−0.2 (4)	C7—N1—S1—O2	165.74 (17)
C12—C7—C8—C9	2.6 (3)	C7—N1—S1—O1	36.48 (19)
N1—C7—C8—C9	−179.8 (2)	C7—N1—S1—C1	−78.70 (18)
C12—C7—C8—C13	−175.8 (2)	C6—C1—S1—O2	−153.98 (17)
N1—C7—C8—C13	1.8 (3)	C2—C1—S1—O2	26.9 (2)
C7—C8—C9—C10	−0.1 (4)	C6—C1—S1—O1	−23.8 (2)
C13—C8—C9—C10	178.3 (3)	C2—C1—S1—O1	157.06 (17)
C8—C9—C10—C11	−1.7 (5)	C6—C1—S1—N1	91.81 (18)
C9—C10—C11—C12	1.2 (5)	C2—C1—S1—N1	−87.33 (18)
C10—C11—C12—C7	1.2 (4)		

### Hydrogen-bond geometry (Å, °)

**Table:**

*D*—H···*A*	*D*—H	H···*A*	*D*···*A*	*D*—H···*A*
N1—H1N···O1^i^	0.86	2.55	3.179 (2)	131
C5—H5···O2^ii^	0.93	2.57	3.229 (3)	129

Symmetry codes: (i) *x*−1, *y*, *z*; (ii) *x*+1/2, −*y*+1/2, *z*+1/2.
